# Monitoring Prehospital Stroke Care in Utah to Assess the Feasibility of Using EMS Data for Surveillance

**Published:** 2009-09-15

**Authors:** Zane Shaeffer, Dorothy Gohdes, Joshua Legler, Peter Taillac, Barbara Larsen

**Affiliations:** St. George’s University School of Medicine. At the time of this study, Mr Shaeffer was affiliated with the Utah Department of Health.; Albuquerque, New Mexico; Utah Department of Health, Salt Lake City, Utah; Utah Department of Health, Salt Lake City, Utah; Utah Department of Health, Salt Lake City, Utah

## Abstract

Many states are developing data systems that use the data elements from the National Emergency Medical Services Information System (NEMSIS) to monitor prehospital stroke care. To explore the feasibility of using emergency medical services data to monitor prehospital stroke care in Utah, the Heart Disease and Stroke Prevention Program and the state emergency medical services agency identified variables that could potentially be used to describe prehospital stroke care and explored the actual data from the first 16 months since inception of a system compatible with NEMSIS. We were able to develop a case definition for possible stroke and to describe modes of response, response times, destination hospitals, and stroke screening practices. Although not all emergency medical services agencies in Utah used the system and the data were not always complete for each stroke case, it was feasible to design a basic surveillance system for prehospital stroke care by using the data.

## Introduction

Since the introduction of thrombolytic therapy for ischemic stroke, public health authorities and clinical facilities have sought effective ways to reach stroke victims rapidly for evaluation and treatment (1,2). Activating emergency medical services (EMS) to transport possible stroke victims and to notify the hospital in advance of the patient's arrival has become a major area of focus for public education efforts to reduce delay (3). The American Heart Association/American Stroke Association (AHA/ASA) convened an expert panel in 2007 to define strategies for EMS in stroke care systems and to suggest specific measures that would reflect rapid EMS activation, response, and transport to an appropriate facility (4). At the same time that recognition of the importance of prehospital stroke care grew, the National Highway Traffic Safety Administration, in cooperation with the National Association of State EMS Directors, began efforts to develop a uniform EMS dataset, the National EMS Information System (NEMSIS) (5). Many states have since begun the process of developing EMS data systems using specific data elements from NEMSIS (6). State public health authorities in Maine recently published their exploration of the possibilities and limitations of using EMS data for cardiovascular surveillance, program planning, and evaluation (7).

In Utah, public and private EMS agencies use the state-owned Prehospital Online Active Reporting Information System (POLARIS) to document the NEMSIS data elements. POLARIS enables EMS professionals to create and submit patient care reports when they provide medical services during emergency responses within the state. POLARIS complies with the NEMSIS data standard. Although EMS agencies in Utah can use any commercial software that is compliant with NEMSIS, POLARIS acts as the state hub that receives data from all agencies. By 2008, 82 of 140 (58.6%) Utah EMS agencies were using an electronic system to submit data. The Utah Heart Disease and Stroke Prevention Program and the Bureau of EMS of the Utah Department of Health collaborated to explore using data from POLARIS to determine dispatch and response modes, response times, stroke screening practices, destination facilities, and other aspects of prehospital stroke care. This article presents information from the cooperative effort between the 2 divisions in Utah exploring the feasibility of using EMS data to monitor prehospital stroke care.

## Methods

In January 2008, the Heart Disease and Stroke Prevention Program reviewed the NEMSIS data dictionary, which includes 424 variables covering agency characteristics, and demographic and clinical information (6). After carefully reviewing the recommendations and measures suggested by the AHA/ASA, we reviewed the list of the 315 NEMSIS data elements that were included in Utah's POLARIS system (3). We identified variables that might be used to define stroke and to address AHA/ASA recommendations. Through this process, the following questions were derived: 1) Are all patients with cases that are identified as possible stroke at time of dispatch or at scene transported as urgent with minimal delays? 2) What are the response times, and do they reflect the minimum feasible time? 3) Are patients with possible stroke screened by EMS and is the duration of symptoms recorded? 4) Are patients with possible stroke cases taken to destination hospitals that are prepared to diagnose and treat stroke promptly?

After discussions about which variables to use, 16 months of data were extracted from the POLARIS database starting at its inception in October 2006 through January 2008. After reviewing the actual data, we refined our definitions and identified both the limitations and the opportunities within the NEMSIS data to describe prehospital stroke care. We decided which NEMSIS code numbers, variables, and definitions to extract from POLARIS to describe prehospital stroke care in Utah (Table).

A possible stroke case was defined as a case in which the EMS personnel's primary impression (E09_15) was stroke or a case that was dispatched as stroke (E03_01) but a primary impression was not listed (suggesting that the EMS personnel agreed with the dispatch diagnosis). To examine the feasibility of using the data to describe response times, we included only possible stroke cases that were both treated and transported to a destination hospital. We then explored variables describing other potential measures of stroke care and stratified the information by location and destination hospital. The total response time was calculated from the time the 9-1-1 call was received, dispatch time, the turnout time (the time from receipt of the dispatch call until the responding unit leaves for the scene), travel time to the scene, and travel time from the scene to the emergency department (ED). We also combined the scene to ED time with the time of symptom onset recorded at the scene to estimate the total time since symptom onset, a key interval for identifying possible eligibility for thrombolytic therapy.

The destination county (E20_06) was used to define a possible stroke case as urban or rural. Urban destinations were the following Utah counties: Salt Lake, Davis, Utah, and Weber; rural denoted all other counties. In urban areas, the 4 destination hospitals recognized as stroke centers were compared with others to determine what percentage of patients with possible stroke cases were being transported to the appropriate hospital. We also ascertained the percentage of patients with possible stroke cases that were transported to the 6 rural hospitals with telestroke capabilities. These hospitals can communicate with the comprehensive stroke center by a real-time audio and video system, allowing the stroke neurologist to "see" and "examine" the patient at a distant hospital.

All data analyses were performed by using SAS version 9.1 (SAS Institute, Cary, North Carolina); dichotomous variables were analyzed for differences using χ^2^, whereas continuous variables, which were nonparametric, were analyzed for differences by Wilcoxon rank-sum test.

## Results

There were 799 cases that met the definition of possible stroke. Of these, 59.7% (n = 477) listed a primary impression of stroke, and the remaining cases (n = 322) were classified as possible stroke because they were dispatched as stroke but had no other diagnosis recorded. Most possible stroke cases (95.9%, n = 766) listed 9-1-1 as the response type. Others were identified as interfacility transfers or medical transports.

Sufficient information to calculate total response times (from 9-1-1 call to arrival at the ED) was available for only 132 of 799 possible stroke cases. The mean response time was 43.5 (SD 33.8) minutes. Information to calculate time from EMS notification to arrival on scene was available for 562 of 799 possible stroke cases; the mean time was 8.8 (SD 6.8) minutes. On-scene time (n = 561) averaged 14.3 minutes. Scene to ED was available for 652 and averaged 19.2 minutes.

Duration of onset (time from symptom onset until queried by EMS) was recorded for 344 of 799 possible stroke cases. Overall, the median duration of onset was 40 minutes. In urban areas, duration of onset was recorded for 57.8% (n = 199) of stroke cases with a median time of 30 minutes. In rural areas, 42.2% of stroke cases (n = 145) had a recorded median time of 45 minutes. The differences in duration of onset between rural and urban areas were not significant (*P* = .38).

There were 298 possible stroke cases that had sufficient data to calculate an estimate of symptom onset to ED arrival. Overall, 69.1% (206 of 298) had a symptom onset to ED arrival time of less than 120 minutes. In urban areas, 70.8% (119 of 168) of possible stroke cases had sufficient information recorded to calculate an onset-to-ED-door time of less than 120 minutes. In rural areas, 66.9% (87 of 130) had an onset-to-ED time of less than 120 minutes.

Determining the use and results of stroke scales proved problematic when the actual data were examined. Very few records indicated that the stroke scale had been performed. On review of the POLARIS data, we found that the stroke scale element was placed in the wrong section and was  overlooked by EMTs filling out patient care reports because of its misplacement.

Of 799 possible stroke cases, 676 had a destination hospital identified. In urban areas, 64.3% (222 of 345) of patients with possible stroke cases were taken directly to 1 of the 4 Joint Commission-certified primary stroke centers in urban Utah. In rural areas, 16.6% (55 of 331) of patients with possible stroke cases were transported to hospitals with telestroke capability.

Of those cases that occurred in urban areas and whose record included a destination hospital, sufficient information was available for 91 cases to calculate total response times (from 9-1-1 call to arrival at the ED). Of these 91 cases, 54 were taken to a certified primary stroke center, and the mean total response time was 50.0 (SD 46.6) minutes. Thirty-seven of the 91 were taken to other urban hospitals with a mean total response time from 9-1-1 call to ED arrival of 33.8 (SD 11.5) minutes.

## Discussion

Prehospital stroke care is a vital link in the stroke system of care. Electronic EMS data that are compatible with the NEMSIS standard can provide important information about transport modes, response times, destinations, and other measures of prehospital stroke care. Collaborative efforts to improve both data collection and measures of prehospital stroke care can benefit both the EMS agencies and state heart disease and stroke prevention efforts.

Using EMS data to describe prehospital stroke care presents formidable challenges. Our definition of possible stroke included those cases dispatched as stroke without actual confirmation that the EMS personnel concurred with the dispatch diagnosis. As did investigators in Maine when they examined cardiac events, we also found that first responders may transfer care to another agency for transport, causing the EMS database to contain separate partial records for the same case (7). For most of this exploratory analysis, we restricted our analyses to records from runs that included both dispatch-to-scene and scene-to-destination information in a single record and did not try to link multiple runs. In addition, electronic EMS data systems are only now being adopted widely, and the process of designing and implementing systems is uneven. In fact, 3 of the largest EMS agencies in the Salt Lake Valley were not submitting NEMSIS-compliant information at the time of the data review. They are all now in the process of developing NEMSIS-compliant systems.

We found that the location of a critical field in the data entry form can determine whether the field is used. POLARIS has now moved the stroke-screen field to a different location in the record. Also, busy EMS providers may not complete all relevant data fields during a run. Specific data about components of response time were lacking on many records.

There are other obvious limitations. All stroke cases may not be diagnosed as stroke in the prehospital setting, and the data reflect care only from areas where EMS agencies use the data collection system. Finally, NEMSIS data elements do not specifically address another important part of stroke care: prehospital notification of stroke (in which the EMS team notifies the hospital that they are bringing in a stroke patient so the hospital can prepare a stroke team to receive the patient on arrival).

The specific findings of this exploratory analysis are limited in that they represent only a fraction of the stroke cases that likely occurred in Utah during the 16-month period covered by the data collection. The number of EMS agencies using POLARIS increased during the period. Nonetheless, this preliminary analysis of the state NEMSIS data demonstrates that important information about stroke care can be monitored using NEMSIS-compatible electronic EMS data.

Many states are adopting NEMSIS-compatible EMS systems, and both cardiovascular health programs and EMS programs can benefit from collaboratively examining state EMS data about prehospital stroke care. The Bureau of EMS of the Utah Department of Health gained information about how completely data were being collected, and it identified the need to reorganize the placement of the field containing the performance and results of formal stroke screening. Response times can be calculated and compared over time as data quality improves and EMS providers recognize the importance of completing the time fields. The Utah Department of Health Heart Disease and Stroke Prevention Program gained insight into new ways to evaluate and improve important measures of prehospital stroke care. Now that designated stroke centers have been developed, EMS personnel must be able to identify the most appropriate destination of possible stroke cases to coordinate timely evaluation and treatment statewide. Thus, this preliminary exploration of NEMSIS data fields confirms that state EMS data can form the basis for a surveillance system for monitoring prehospital stroke care, an essential component of the stroke system of care envisioned by the AHA/ASA and its many partners.

## Figures and Tables

**Table.** National Emergency Medical Services Information System Codes Used to Enhance Stroke Surveillance System

**NEMSIS Code**	**Title**	**Definition**
**E02_02**	Incident number	The incident number assigned by the 9-1-1 dispatch system.
**E02_04**	Type of service requested	The type of service or category of service requested of the EMS responding to this specific EMS incident.
**E03_01**	Complaint reported by dispatch	The complaint that dispatch reported to the responding unit.
**E05_02**	Public Safety Answering Point (9-1-1 call center) call date/time	The date and time the phone rings (9-1-1 call to public safety answering point or other designated entity) requesting emergency medical care.
**E05_03**	Dispatch notified date/time	The date and time dispatch was notified by the 9-1-1 call taker (if a separate entity).
**E05_04**	Unit notified by dispatch date/time	The date and time the responding unit was notified by dispatch.
**E05_07**	Arrived at patient date/time	The date and time the responding unit arrived at the patient's side.
**E05_09**	Unit left scene date/time	The date and time the responding unit left the scene (started moving).
**E05_10**	Patient arrived at destination date/time	The date and time the responding unit arrived with the patient at the destination or transfer point.
**E09_06**	Duration of chief complaint	The time duration of the chief complaint.
**E09_07**	Time units of duration of chief complaint	The time units of the duration of the patient's chief complaint.
**E09_15**	Providers' primary impression	The EMS personnel's impression of the patient's primary problem or most significant condition that led to the management given to the patient (treatments, medications, or procedures).
**E14_24**	Stroke scale	The patient's Los Angeles or Cincinnati Stroke Scale Results.
**E20_01**	Destination/transferred to, name	The destination to which the patient was delivered or transferred.
**E20_06**	Destination county	The destination county to which the patient was delivered or transferred.
**E20_10**	Incident/patient disposition	Type of disposition treatment or transport of the patient, or both.

Abbreviations: EMS, emergency medical service.

Source: National Emergency Medical Services Information System (5).
